# Hearing loss in humans drinking tube well water with high levels of iron in arsenic–polluted area

**DOI:** 10.1038/s41598-019-45524-1

**Published:** 2019-06-21

**Authors:** Tingchao He, Nobutaka Ohgami, Xiang Li, Ichiro Yajima, Reina Negishi-Oshino, Yoko Kato, Kyoko Ohgami, Huadong Xu, Nazmul Ahsan, Anwarul Azim Akhand, Masashi Kato

**Affiliations:** 1 Department of Occupational and Environmental Health, Nagoya University Graduate School of Medicine, Nagoya, Japan; 2 Voluntary Body for International Health Care in Universities, Nagoya, Japan; 3 Department of Genetic Engineering and Biotechnology, University of Dhaka, Dhaka, 1000 Bangladesh

**Keywords:** Epidemiology, Epidemiology, Auditory system

## Abstract

Well water for drinking with increased levels of iron in arsenic-polluted areas has been reported worldwide. Oral exposure to arsenic has been shown to be associated with hearing loss, while there is no evidence for an association between excessive exposure to iron and hearing loss in humans. In this study, we determined iron and arsenic levels in biological samples and hearing levels by pure tone audiometry (PTA) in subjects in a control area and an arsenic-polluted area in Bangladesh. The iron level in well water in the arsenic-polluted area was significantly higher than that in piped supply water in the control area. Subjects in the polluted area (n = 109), who had higher iron and arsenic levels in hair and toenails than those in subjects in the control area (n = 36), had an increased risk of hearing loss at 8 kHz and 12 kHz after adjustments for age, gender, smoking and BMI. Significant associations of the exposure group with hearing loss at 8 kHz and 12 kHz remained after further adjustment for arsenic levels in toenails and hair. Thus, this pilot study showed that excessive exposure to iron via drinking water is a potential risk for hearing loss in humans.

## Introduction

Well water for drinking that is contaminated with toxic elements including arsenic has been reported worldwide^1–3^. It was estimated that about 20 million Bangladeshi are orally exposed to arsenic via drinking well water^4^. High levels of iron as well as arsenic have been reported in drinking well water in some countries including Bangladesh^1^, Vietnam^5^ and the United States^6^. In a previous study, tube wells with high iron and low arsenic levels and those with low iron and high arsenic levels were also found in several areas in Bangladesh^7^. Thus, it is possible that people are exposed to not only arsenic but also iron by drinking tube wells.

Excess exposure to iron has been shown to be associated with health risks including diabetes, obesity, non-alcoholic fatty liver disease and Alzheimer’s disease in humans^8–10^. An experimental study showed that elevated iron levels in serum exacerbated aminoglycoside ototoxicity in pigmented guinea pigs compared with the controls only receiving gentamicin^11^. Another study showed that insulin resistance due to dietary iron overload reduced the number of ribbon synapses that led to auditory brain response (ABR) threshold elevations^12^, suggesting that excess exposure to iron might affect hearing levels in experimental animals. In addition, a case-report study indicated that hemochromatosis, which is characterized by excessive absorption and storage of iron from the diet, affected functional hearing ability^13^. It was also shown by longitudinal analysis that a patient with superficial hemosiderosis of the central nervous system developed bilateral sensorineural hearing loss^14^. There is no information about the association between excessive exposure to iron in drinking water and hearing loss in humans, although oral exposure to arsenic has been shown to be associated with hearing loss in humans^15^.

In this study, we compared iron and arsenic levels in toenails and hair in a control group drinking piped supply water and an exposure group drinking tube well water contaminated with arsenic in Bangladesh. We then investigated the association of hearing loss with exposure to iron using multivariate analysis adjusted for age, sex, body mass index (BMI), smoking and arsenic levels in the biological samples to determine whether exposure to iron is a risk factor for hearing loss in humans.

## Methods

### Study population

Participants were recruited from the control area (control group) and the arsenic-polluted area (exposure group) in Bangladesh by convenience sampling. The control and arsenic-polluted areas are located in urban and rural areas, respectively. All of the participants received education. After excluding subjects according to predetermined exclusion criteria including a) ear-related disease history or other diseases at the time of our study and b) a history of cosmetic hair and toenail application, a total of 145 subjects aged from 12 to 55 years including housewives, businessmen and students were included in our final analysis. There were no regular alcoholic drinkers or portable music player listeners among the subjects. Information was obtained from questionnaires that included questions on age, gender, smoking, clinical history, and duration of drinking tube well water. Trained investigators were in charge of filling in the questionnaires by asking questions, and quality inspection was also performed during field work. Nagoya University International Bioethics Committee (approval number 2013-0070) and Faculty of Biological Science, University of Dhaka (Ref. no. 5509/Bio.Sc) reviewed and approved the research, and all research was performed in accordance with relevant guidelines/regulations. All of the participants signed a written informed consent form for participation in the study.

### Hearing examination by pure tone audiometry (PTA)

Our method for hearing examination has been described and published elsewhere^15–19^. Briefly, we measured hearing levels by PTA at 1, 4, 8 and 12 kHz to determine the auditory threshold of subjects at each frequency. The examination was performed with an iPod and headphones (earphone type, Panasonic RP-HJE150) in a soundproof environment created with earmuffs with a noise reduction rating of 25 dB^20^. The operator stood just behind each subject. The examination was started by giving each subject an initial sound stimulus of 5 dB at 1 kHz and the stimuli were then increased by 5 dB stepwise. When the subjects recognized the sound, they raised their hands. The operator recorded the sound level recognized by each subject as the hearing threshold. Output pure tones at each frequency were verified with a noise level meter (Type 6224 with an FFT analyzer, ACO CO., LTD, Japan). We performed the test twice for both ears of each participant for repeatability of results. We used hearing levels in the left ear of each subject for multivariate analyses since hearing levels of both ears were comparable.

### Measurement of iron and arsenic levels in drinking water samples and biological samples

The method for collection and measurements of drinking water samples followed a previous report^21^. Subjects in the control group (n = 36) used the same source of piped supply water for drinking water, while subjects in the exposure group (n = 109) shared four tube wells for drinking water that were located near their houses in a village in their daily life and stated that they did not drink tube well water from another source. Drinking water samples were therefore taken from the four tube wells. Four water samples were also randomly collected from the household piped supply water of the 36 subjects in the control group. The reason why only 36 subjects were included as control subjects was that we could identify the source of drinking water used in their daily life. Household piped supply water was collected from houses of only four randomly selected participants since the source of piped supply water was the same, since they used piped supply water and its original source was the same. The methods for collection and measurements of hair and toenail samples followed previous studies^15,18,22,23^. Briefly, hair and toenail samples were obtained from the occipital area and toe, respectively, and they were immediately stored in 15 ml polypropylene tubes with 3 ml 61% nitric acid after clipping. The tubes were incubated at 80 °C for 48 h, and 3 ml of 30% hydrogen peroxide solution was added to each tube after cooling down at room temperature for 1 h. Then the tubes were incubated at 80 °C for 3 h. Finally, the samples were diluted with ultrapure water and measurements were carried out by using an inductively coupled plasma mass spectrometer (ICP-MS; 7500cx, Agilent Technology).

### Definitions and classification

Hearing losses at 1, 4, 8 and 12 kHz were defined as ≥10 dB, ≥10 dB, ≥25 dB and ≥40 dB, respectively, according to a previous study^16^. The height and weight of each subject were self-reported on their previous physical measurement record. Body mass index (BMI) was calculated by weight in kilograms divided by the square of height in meters and was classified into underweight (<18.5 kg/m^2^), normal range (18.5 ~ 25 kg/m^2^), and overweight or obesity (≥25 kg/m^2^) on the basis of the WHO classification^24^.

### Statistical analyses

Quantitative variables with a normal distribution are presented as means ± standard deviations (SD) or otherwise presented as medians and interquartile ranges (IQRs). Categorical variables are presented as numbers and percentages. The Mann-Whitney *U* test was used to investigate differences of iron and hearing levels between the control and exposure groups. Finally, we used a logistic regression model to investigate the association between drinking tube well water and auditory threshold. Descriptive and relationship analyses were carried out by using the Statistical Package for Social Sciences Version 24.0 (SPSS Inc., Chicago, IL, USA). All statistical tests were 2-sided and a *P* value < 0.05 was considered to be statistically significant.

## Results

### Iron and arsenic levels in drinking water and characteristics of subjects

**Figure 1:**
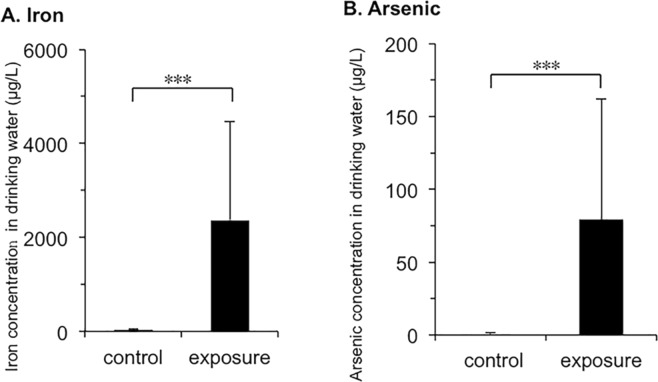
Iron and arsenic concentrations in drinking water in the control and arsenic-polluted areas. Iron and arsenic levels (means ± SD) in tube well water in the arsenic-polluted area (exposure; n = 4) and in piped supply water in the control area (control; n = 4) were measured. A significant difference (***P < 0.001) was determined by the Mann-Whitney *U* test. (**A**) Iron levels in tube well water and piped supply water. (**B**) Arsenic levels in tube well water and piped supply water.

**Figure 2:**
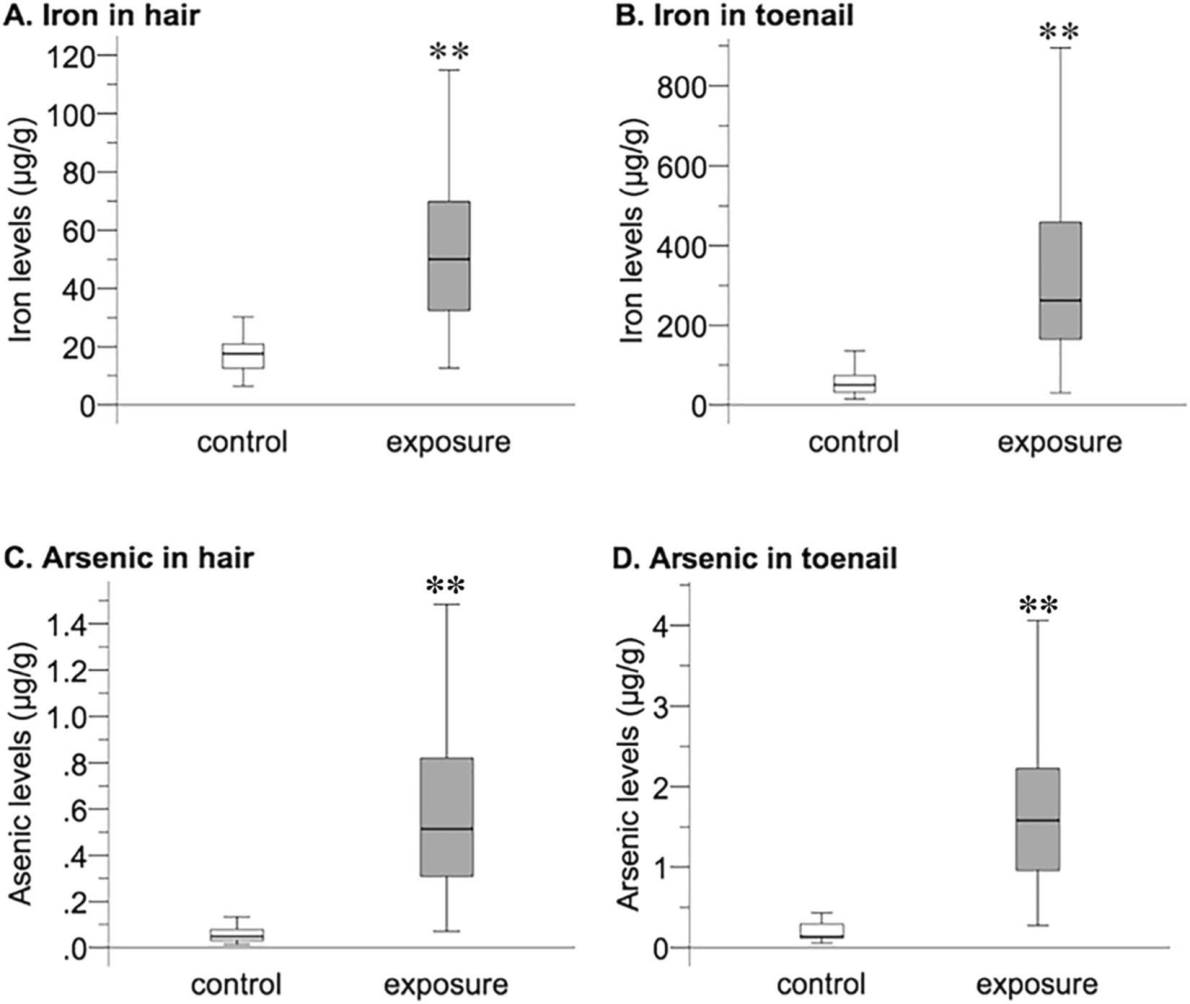
Iron and arsenic levels in biological samples from subjects in the control and exposure groups. Iron and arsenic levels (medians, interquartile ranges) in hair and toenail samples from subjects in the exposure group (n = 109) and control group (n = 36) are shown. A significant difference (**P < 0.01) was determined by the Mann-Whitney *U* test. (**A**) Iron in hair. (**B**) Iron in toenails. (**C**) Arsenic in hair. (**D**) Arsenic in toenails.

The iron concentration in drinking well water (2,367.6 ± 2,094.7 μg/L) was significantly higher than that in piped supply water (29.8 ± 27.1 µg/L). The arsenic concentration in drinking well water (79.4 ± 82.8 µg/L) was also significantly higher than that in piped supply water (0.6 ± 0.7 µg/L) (Fig. 1). Characteristics of the subjects in this study are shown in Table 1. Subjects in the control group were younger than those in the exposure group, and the exposure group had larger proportions of females and smokers (all *P* values < 0.01). There was no significant difference in BMI between the two groups. The median iron concentrations in hair and toenail samples were 17.6 µg/g and 50.1 µg/g (Fig. 2A), respectively, in the control group and 50.0 µg/g and 262.6 µg/g, respectively, in the exposure group (Fig. 2B). The median concentrations of arsenic in hair and toenail samples were 0.05 µg/g and 0.4 µg/g (Fig. 2C), respectively, in the control group and 0.5 µg/g and 1.6 µg/g, respectively, in the exposure group (Fig. 2D). Both iron and arsenic levels in the exposure group were significantly higher than those in the control group for all biological samples (*P* < 0.001) (Fig. 2).

**Table 1:** Characteristics of subjects in the study.

Characteristics		Control	Exposure	Total	*P* value
		(n = 36)	(n = 109)	(n = 145)	
Age		23.8 ± 5.4	31.5 ± 11.7	29.58 ± 10.96	<0.001
BMI		22.6 ± 2.7	21.8 ± 3.6	21.99 ± 3.43	0.125
Gender	Male	31 (86.1)	38 (34.9)	69 (34.9)	<0.001
	Female	5 (13.9)	71 (65.1)	76 (52.4)	
Smoking	No	34 (94.4)	80 (73.4)	114 (78.6)	0.008
	Yes	2 (5.6)	29 (26.6)	31 (21.4)	

BMI: body mass index.

Data are expressed as means ± standard deviations for continuous variables with a normal distribution and as numbers (percentages) for categorical variables.

### Univariate analysis of hearing loss in the exposure group

**Figure 3:**
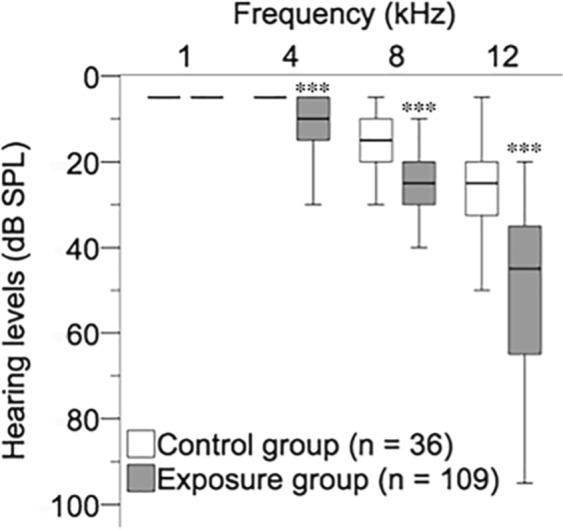
Auditory thresholds of subjects in the control and exposure groups. Hearing thresholds (medians, interquartile ranges) in the exposure group (n = 109) and control group (n = 36) at 1 kHz, 4 kHz, 8 kHz and 12 kHz are shown. A significant difference (***P < 0.001) was determined by the Mann-Whitney *U* test.

Hearing levels in the control and exposure groups are shown in Fig. 3. The median auditory thresholds at 4, 8, and 12 kHz in the exposure group were 10, 25, and 40 dB, respectively, and they were significantly higher than those in the control group, 5, 15 and 25 dB, respectively (P < 0.001). There was no significant difference in the auditory threshold at 1 kHz between the two groups.

### Multivariate analysis of iron-mediated hearing loss in the exposure group

Compared with the control group, significant associations between the exposure group and hearing loss were found at 8 kHz (OR = 6.994; 95% CI: 1.959, 24.974; *P* = 0.003) and 12 kHz (OR = 8.574; 95% CI: 2.507, 29.327; *P* < 0.001) after adjustments for age, gender, BMI and smoking (Table 2), while there was no significant difference at 1 kHz (*P* = 0.805) or 4 kHz (*P* = 0.058). There were significant associations between hearing loss and the exposure group at 4 kHz (OR = 7.592; 95% CI: 2.745, 20.994; *P* < 0.001), 8 kHz (OR = 13.800; 95% CI: 4.548, 41.875; *P* < 0.001) and 12 kHz (OR = 13.109; 95% CI: 4.696, 36.593; *P* < 0.001) without adjustment (Table 2). In our previous animal and epidemiology study, arsenic was found to be associated with hearing loss, and hair and toenail samples were shown to reflect the status of chronic exposure to arsenic^15^. Hence, we additionally adjusted for arsenic in both hair and toenail samples, as shown in Table 3. There were significant associations between the exposure group and hearing loss at 8 kHz (OR = 5.555; 95% CI: 1.440, 21.437; *P* = 0.013) and 12 kHz (OR = 3.928; 95% CI: 1.011, 15.261; *P* = 0.048) but not at 4 kHz (OR = 2.228; 95% CI: 0.599, 8.284; *P* = 0.232) in hair samples. In the model, the exposure group was also associated with hearing loss at 4 kHz (OR = 4.860; 95% CI: 1.257, 18.785; *P* = 0.022), 8 kHz (OR = 8.301; 95% CI: 2.059, 33.457; *P* = 0.003) and 12 kHz (OR = 5.316; 95% CI: 1.376, 20.543; *P* = 0.015) in toenail samples.

**Table 2:** Odds ratios (95% *CI*) of hearing loss in the exposure group.

	Hearing loss			
	1 kHz	4 kHz	8 kHz	12 kHz
	(≥10 dB)	(≥10 dB)	(≥25 dB)	(≥40 dB)
**Model 1**				
Control	Reference	Reference	Reference	Reference
Exposure	1.646 (0.619, 4.380)	7.592^***^ (2.745, 20.994)	13.800^***^ (4.548, 41.875)	13.109^***^ (4.696, 36.593)
*P* value	0.318	<0.001	<0.001	<0.001
**Model 2**				
Control	Reference	Reference	Reference	Reference
Exposure	0.854 (0.243, 2.994)	3.236 (0.962, 10.893)	6.994^**^ (1.959, 24.974)	8.574^**^ (2.507, 29.327)
*P* value	0.805	0.058	0.003	<0.001

Abbreviation: CI, confidence interval.

Reference: OR = 1.

Model 1: preliminary model without adjustment.

Model 2: adjustments for age, gender, smoking, and BMI, which have been reported to affect hearing.

*P* value was determined by binary logistic regression analysis, **P < 0.01, ***P < 0.001.

**Table 3:** Odds ratios (95% *CI*) of hearing loss in the exposure group with adjustment for arsenic in biological samples.

	Hearing loss			
	1 kHz	4 kHz	8 kHz	12 kHz
	(≥10 dB)	(≥10 dB)	(≥25 dB)	(≥40 dB)
**Adjusted for arsenic in hair** ^**a**^				
Control	Reference	Reference	Reference	Reference
Exposure	0.675 (0.174, 2.612)	2.228 (0.599, 8.284)	5.555* (1.440, 21.437)	3.928* (1.011, 15.261)
*P* value	0.569	0.232	0.013	0.048
**Adjusted for arsenic in toenails** ^**a**^				
Control	Reference	Reference	Reference	Reference
Exposure	0.936 (0.226, 3.881)	4.860* (1.257, 18.785)	8.301** (2.059, 33.457)	5.316* (1.376, 20.543)
*P* value	0.927	0.022	0.003	0.015

Abbreviation: CI, confidence interval.

Reference: OR = 1.

^a^The models were also adjusted for age, gender, smoking, BMI, which were reported to affect hearing.

*P* value was determined by binary logistic regression analysis, *P < 0.05, **P < 0.01.

## Discussion

This study demonstrated that iron and arsenic concentrations in drinking water in the arsenic-polluted area were significantly higher than those in the control area. The study also showed that levels of iron and arsenic in hair and toenail samples from subjects in the exposure group were significantly higher than those in samples from subjects in the control group. Multivariate analysis with adjustments for age, sex, BMI and smoking showed associations between the exposure group and hearing loss. Thus, this study demonstrated hearing loss in humans drinking tube well water with high levels of iron in an arsenic-polluted area.

In previous studies, levels of metal elements in blood samples^25^ and urine samples^26^ were shown to reflect the status of acute exposure to the metal elements, while nail and hair samples were used to evaluate the status of chronic exposure to the metal elements^27,28^. In our previous study, multivariate analysis showed that significant associations of arsenic levels in toenails with heating loss were at 4, 8 and 12 kHz, while a significant association of arsenic levels in hair with hearing loss was limited to 12 kHz^19^. Hearing levels at 4 kHz and 8 kHz are known to be important for daily conversation and examinations of noise-induced hearing loss and age-related loss, respectively, while 12 kHz is an extra-high frequency and hearing ability at 12 kHz is not necessary for daily communication in humans. Based on the information above, arsenic levels in toenails were used in this study as confounders in the models to further determine the effect of arsenic on the odds ratios of hearing loss at 4 kHz and 8 kHz in the exposure group with high iron levels as well as high arsenic levels in toenails. Arsenic levels in hair were also used in this study as confounders in the models to further determine the effect of arsenic on the odds ratios of hearing loss at 12 kHz in the exposure group with high iron levels as well as high arsenic levels in hair. In the multivariate analysis, the significant associations between the exposure group and hearing loss at 4, 8 and 12 kHz remained after adjustment for arsenic in toenails, while the significant associations with hearing loss at 8 and 12 kHz remained after adjustment for arsenic in hair. Thus, our study suggests that exposure to iron by drinking well water is an independent risk factor for hearing loss in humans. In this study, we found that there was a significant correlation of iron levels in toenails with duration of drinking well water (Fig. S1). In our previous study, a significant correlation of arsenic levels in toenails with duration of drinking well water was also shown^3^. We therefore determined iron and arsenic levels in toenails in different groups living in different areas in Bangladesh as a separate investigation to determine the individual risk of excess exposure to iron, but not arsenic, on hearing loss (Fig. S2). A high iron group (n = 99) had a significantly higher level of iron in toenails than that in a low iron group (n = 50) (Fig. S2A), although arsenic levels in toenails in the two groups were comparable (Fig. S2B) and similar to that in the control group in this study (Fig. 2D), although we do not have exact information about exposure routes in the additional two groups. We next compared hearing levels at 4 kHz in the two groups (Fig. S2C), since hearing level at 4 kHz is known to be important for daily communication. The high iron group (n = 99) had a worse hearing level at 4 kHz than that in the low iron group (n = 50) (Fig. S2C). Furthermore, multivariate analysis adjusted for age, BMI and smoking showed a significant risk of hearing loss in the high iron group (Table S1). Thus, our additional data also suggest an individual risk of excessive exposure to iron associated with hearing loss.

In this study, the average iron level in drinking well water (2,367.6 ± 2,094.7 μg/L) exceeded the iron concentration standard for drinking water of Bangladesh, which ranges from 0.3 to 1.0 mg/L^29^. In previous studies, iron concentrations in drinking well water in Bangladesh were shown to be higher than 2.0 mg/L in 72% of tube wells (948 wells) in a rural northwestern area^30^, and levels ranged from 1.03 mg/L to 24.50 mg/L in Tangail City in the central region of Bangladesh^31^. According to a national drinking water quality survey in Bangladesh, the average concentrations of iron in drinking water reached 2.22 mg/L^29^. On the other hand, the average iron level in drinking well water in India has been shown to be 2.2 mg/L^32^ with a maximum concentration of 51 mg/L^33^. Iron levels ranging from 0.15 to 25 mg/L in well water in the western area of the USA were also reported^34^. Thus, it is possible that countries other than Bangladesh also have high concentrations of iron in drinking water, although iron concentrations in groundwater varied depending on geographical or aquifer features, the depth of the tube well and the sampling season. Iron levels in hair and toenails in this study were similar to those in previous studies, in which they ranged from 13.5 µg/g to 300 µg/g in hair^35^ and from 9.16 µg/g to 135 µg/g in nails^32^. Further investigation is needed to determine the associations between hearing loss and exposure to iron via drinking well water worldwide.

In this study, we assessed the regression model’s goodness of fit and each variable’s contribution in the model at each frequency with Nagelkerke R^2^ and Pseudo R^2^ values, respectively^36,37^. The values of Nagelkerke R^2^ and Pseudo R^2^ range from 0% to 100%, with higher values indicating higher goodness of the model and higher contribution of each variable in the model (Table S4). For this analysis, we divided the subjects into two groups with cut-off values of iron levels in toenails and hair (Table S2) obtained by receiver operating characteristic (ROC) curves and the Youden index^19^ in order to compare the hearing levels between the two groups (Fig. S2 and Table S3). Univariate analysis and multivariate analysis with adjustment for age, gender, smoking, and BMI showed that high iron levels in hair and toenails were significantly associated with hearing loss at 4, 8 and 12 kHz (Fig. S2 and Table S3). The relative contributions of iron (10.77% in hair and 7.32% in toenails) to hearing loss at 4 kHz were higher than those of arsenic (6.59% in hair and 1.11% in toenails) (Table S4). Likewise, the relative contributions of iron (11.26% in hair and 6.95% in toenails) to hearing loss at 8 kHz were higher than those of arsenic (5.77% in hair and 0.26% in toenails), while those of iron (6.98% in hair and 4.04% in toenails) at 12 kHz were less than those of arsenic (24.15% in hair and 11.90% in toenails) (Table S4). Thus, our multivariate analysis suggests that iron is a greater contributor than arsenic to hearing loss at 4 and 8 kHz in these models, while arsenic is a greater contributor than iron to hearing loss at 12 kHz. Age is known to be a strong factor for hearing loss. Correspondingly, age was the greatest contributor among confounders to hearing loss at 4, 8 and 12 kHz in these models (Table S4). In addition, the interaction effect of iron and arsenic levels in toenails and hair was determined in the logistic regression models to determine the synergistic influence of iron and arsenic on hearing loss (Table S5). There were no significant interaction effects of iron and arsenic on hearing loss at least in the models (Table S5). Therefore, it is not likely that there is synergistic influence of co-exposure to iron and arsenic on hearing loss in humans, although a previous study showed synergistic influence of co-exposure to iron and arsenic on carcinogenicity *in vitro*^1^.

Previous studies showed that elements in hair and nails were indexes reflecting chronic exposure status in humans^3,15,18^ since growth of hair and nails generally takes several months. Thus, it is possible that chronic exposure to iron is associated with hearing loss in humans. The mechanism of hearing loss associated with excess exposure to iron is unclear. In previous experimental studies, excess cellular levels of iron were shown to increase reactive oxygen species (ROS) via the Fenton reaction^38,39^. ROS have been shown to cause morphological damage to outer and inner hair cells in the organ of Corti^40,41^. Thus, it is possible that chronic exposure to iron increases ROS, consequently resulting in hearing loss. On the other hand, a significant correlation of an iron-related molecule in urine with that in serum has been shown^42^. In this study, we therefore determined the iron levels in urine samples from male subjects in the control (n = 32) and exposure groups (n = 37), instead of blood samples, since blood samples contain red blood cells with high levels of iron strongly correlated with anemia and we did not have information on the female participants’ menstruation. The iron level in urine samples from male subjects in the exposure group was significantly higher than that in male subjects in the control group (Fig. S4), while multivariate analysis adjusted for age, smoking, BMI and arsenic in urine samples did not show a significant association of iron level in urine samples with hearing loss (Table S6). These results partially correspond to the results in our previous studies showing that toxic elements in toenail samples reflecting chronic exposure status, but not in urine samples, had a significant association with hearing loss^15–19^. In our experimental study as well, chronic exposure, but not acute exposure, to toxic elements via drinking water was shown to cause accumulation of toxic elements in inner ears, resulting in degeneration of auditory nerves and hearing loss in mice^15,43^. Thus, it is possible that hearing loss caused by excessive exposure to toxic elements via drinking water requires chronic exposure rather than acute exposure.

A possible mechanism to explain the above results showing that hearing loss was not associated with acute exposure to iron is as follows. The cellular concentration of iron is known to be strictly regulated by iron homeostasis molecules in order to avoid cellular damage by reactive oxygen species (ROS) induced by increased iron levels. The iron transporters (DMT1, Zip8, Zip14) for iron influx and the iron exporter (ferroportin: FPN) for iron efflux have been shown to mediate iron homeostasis. A previous study demonstrated that the iron transporters (DMT1, Zip8, Zip14) are expressed in the organ of Corti in inner ears of rats^44^. Another study demonstrated a significant association of *FPN* with increased risk of developing sudden hearing loss in humans^45^. Thus, it is possible that the cellular concentration of iron is maintained by iron homeostasis molecules expressed in the organ of Corti in order to avoid an abnormal iron increase in the organ of Corti by acute exposure to iron. On the other hand, a previous study showed that chronic exposure of rats to iron increased lipid peroxidation in the intestine presumably because of increased uptake of iron^46^. Therefore, it is likely that chronic exposure to iron causes impairment of iron homeostasis in the inner ears.

A previous study showed that the mean absorption rate of iron from the gut is 18.8% from water^47^, although the absorption rate of iron depends on the body iron requirements and the chemical forms of iron. In this study, the iron level (mean ± SD) in drinking well water for the exposure group was 2367.6 ± 2094.7 µg/L. If the general amount of drinking water for an adult per day is regarded as 1 L, the total amount of iron contained in well water ingested per day is 2367.6 µg. Thus, the iron amount absorbed from the gut from well water in the exposure group would be about 400 µg per day based on the average amount of total iron contained in well water ingested per day and the absorption rate (18.8%) mentioned above. Although the amount of iron (about 2.4 mg/day) contained in well water in this study is less than the dose of iron (20 mg/day) used for anemia patients for 4–8 months in a previous study^48^, a previous study showed that the national prevalence of iron deficiency in Bangladesh was low (only 9.4% of subjects drinking well water with high iron) contrary to the widely held assumption^49^. Therefore, it is likely that prevalence of anemia is not high in the exposure area in this study. In a future study, it will be necessary to further investigate the issue of anemia with serum samples in the exposure group. According to the national micronutrients survey 2011–2012, daily dietary iron intake is 5.3–7.7 mg/day. If we combine the iron data from dietary intake with our iron data from well water (2.4 ± 2.1 mg/day), the total iron intake of participants in this study is about 7.7–10.1 mg/day. Thus, the iron intake from drinking well water accounts for 23–31% of total iron intake per day, although we did not investigate the dietary intake of each participant on a daily basis. The recommended dietary allowance (RDA) of iron is 8–18 mg/day in a population aged more than 9 years for both genders (except pregnant females)^50,51^. In this study, it is possible that the intake of iron exceeded the RDA in about 8.4% of the female participants and 86.8% of the male participants in the exposure group.

There are several limitations of our study. First of all, our cross-sectional analysis was useful for outlining the association between high iron status and hearing loss, but it could not establish a causal relationship. Cohort studies exploring the causality and studies addressing the underlying pathophysiological mechanisms are still needed. Secondly, there was no information about noise background. Although the levels of daily noise exposure were unclear, it was thought that there was no difference in exposure levels in surrounding environments since the subjects were recruited from a non-occupational noise exposure population including students, housewives, and businessmen. Thirdly, we did not investigate dietary iron intake of participants and iron levels in blood samples. Fourthly, an iPod with headphones was used as the screening method for the field work in Bangladesh, since no clinical audiometer was available in rural areas. In addition, otoscopy and tympanometry were not performed to check middle-ear problems. Finally, the study included a relatively small number of subjects in Bangladesh. Therefore, there is a possibility of bias of the sample size in this pilot study. Further study is needed to verify the findings in this study with a larger number of subjects. Previous studies showed that iron levels in drinking well water exceeded the environmental standards for drinking water in several countries^1,5,6^. Our study suggested that more attention should be paid to this overlooked public issue.

## Conclusion

Our study provided epidemiological evidence that exposure to iron at an excessive level by drinking well water is an independent risk of hearing loss in humans.

## Supplementary information

Supplementary file
