# Supplementary material for: Hearing loss in humans drinking tube well water with high levels of iron in arsenic–polluted area
Source: Sci Rep. 2019 Jun 21;9:9028. doi: 10.1038/s41598-019-45524-1 (PMC6588562; doi:10.1038/s41598-019-45524-1)
Supplement: Supplementary file 1 — Supplementary file [file 41598_2019_45524_MOESM1_ESM.pdf]

Supplementary information

Hearing loss in humans drinking tube well water with high levels of iron in arsenic–polluted area

Tingchao He[a,b], Nobutaka Ohgami[a,b], Xiang Li[a,b], Ichiro Yajima[a,b], Reina Oshino[a,b], Yoko Kato[a,b], Kyoko Ohgami[a,b], Huadong Xu[a,b], Nazmul Ahsan[b,c], Anwarul Azim Akhand[b,c], Masashi Kato[a,b,*]

[a]Department of Occupational and Environmental Health, Nagoya University Graduate School of Medicine, Nagoya, Japan.

[b]Voluntary Body for International Health Care in Universities, Nagoya, Japan.

[c]Department of Genetic Engineering and Biotechnology, University of Dhaka, Dhaka-1000, Bangladesh.

*Corresponding author:

Masashi Kato, M.D., Ph.D.

Department of Occupational and Environmental Health,

Nagoya University Graduate School of Medicine.

Address: 65 Tsurumai-cho, Showa-ku, Nagoya, Aichi 466-8550, Japan.

Phone: +81-52-744-2122. Fax: +81-52-744-2124.

E-mail: katomasa@med.nagoya-u.ac.jp

Figure S1. Correlations of duration of drinking well water with iron levels in toenails of subjects. Correlations of duration of drinking well water with iron levels in toenails in participants determined by Spearman's rank correlation coefficient are presented.

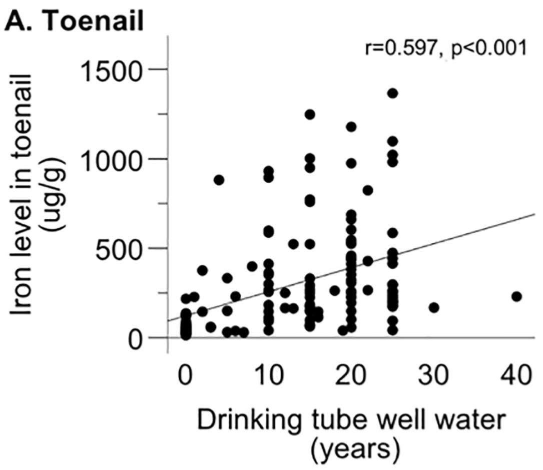

Figure S2. Iron and arsenic levels in toenails and hearing levels in different groups living in different areas of Bangladesh as a separate investigation. Additional analysis was performed from a separate investigation in which male participants were selected in Bangladesh. According to iron levels in toenails, participants were classified into high and low iron groups by receiver operating characteristic (ROC) analysis (Ohgami et al., 2018). The two groups had similar noise background levels and noise levels in both groups were less than noise standard (85dB) (CDC, 1998) in daily and occupational environments. Ages (means ± SD) were 34.6 ± 9.9 and 37.2 ± 11.7 and BMIs (means ± SD) were 23.2 ± 3.0 and 23.2 ± 3.7 in the low and high iron groups, respectively, and there was no significant difference between the two groups. Iron, arsenic and hearing thresholds at 4 kHz (medians, interquartile ranges) in the low iron group (n=50) and high iron group (n=99) are shown. A: Iron in toenails. B: Arsenic in toenails. C: Auditory thresholds of subjects in the high and low groups. A significant difference (***P < 0.001) was determined by the Mann-Whitney *U* test.

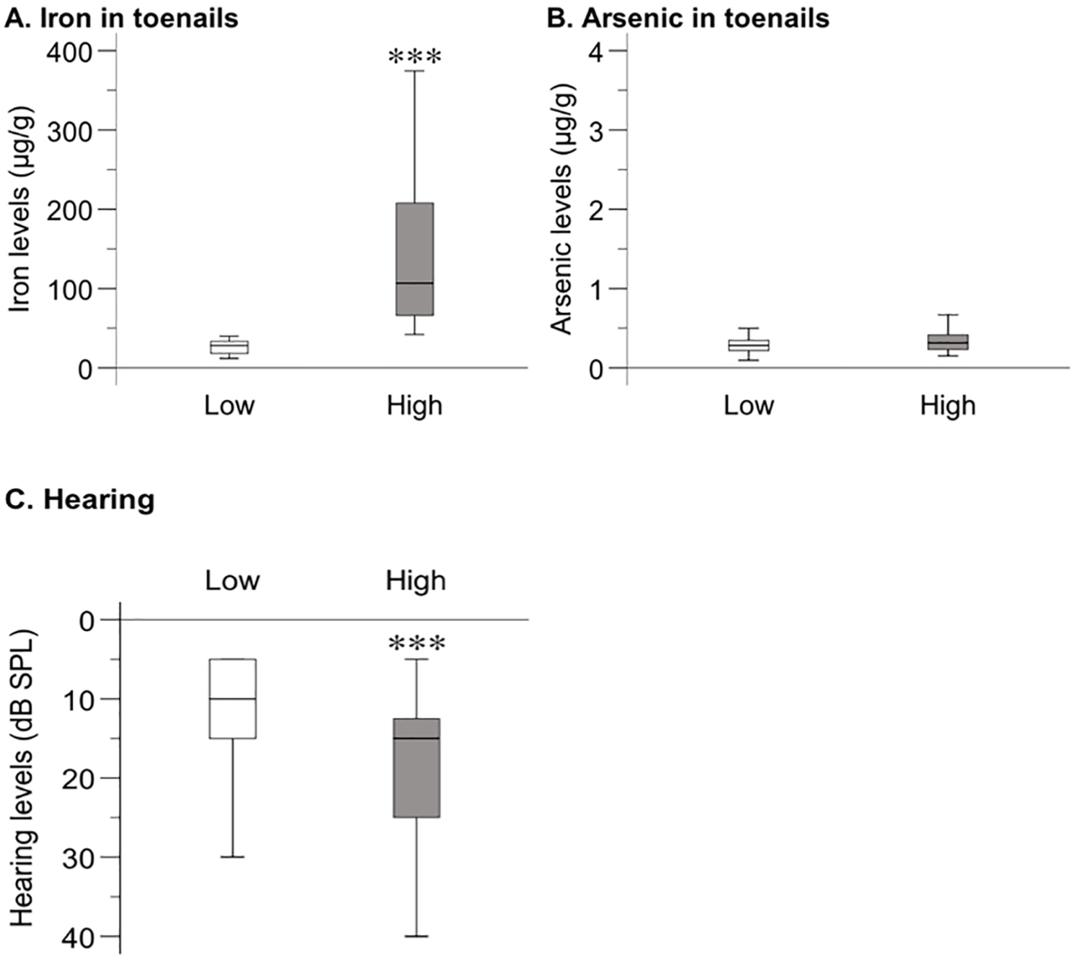

Figure S3. Auditory thresholds of subjects in the high and low iron groups. Hearing thresholds (medians, interquartile ranges) from frequencies of 1 kHz to 12 kHz in the group with low iron levels and the group with high iron levels in hair (A) and toenails (B) are shown. Cut-off values of iron and the number of subjects in each group of the biological samples are shown in Table S1. A significant difference (**P* < 0.05; ***P* < 0.01; ****P* < 0.001) was determined by the Mann-Whitney U test.

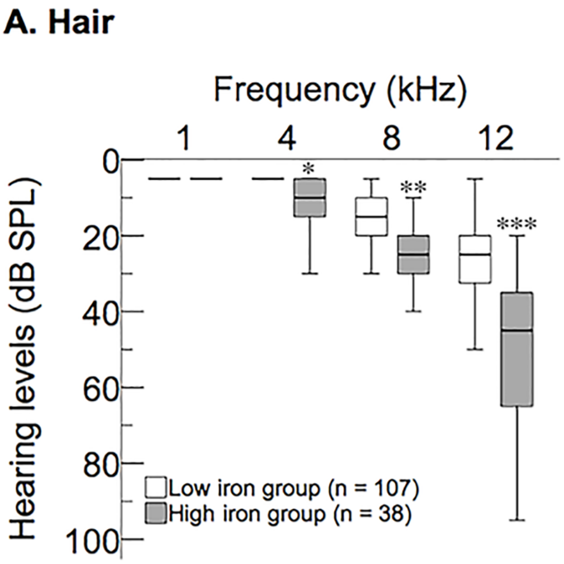

Figure S4. Iron and arsenic levels in urine from subjects in the control and exposure groups. Iron and arsenic levels (medians, interquartile ranges) in urine from male subjects in the exposure group (n=32) and control group (n=37) are shown. A significant difference (**P < 0.01, ***P < 0.001) was determined by the Mann-Whitney U test. A: Iron in urine. B: Arsenic in urine.

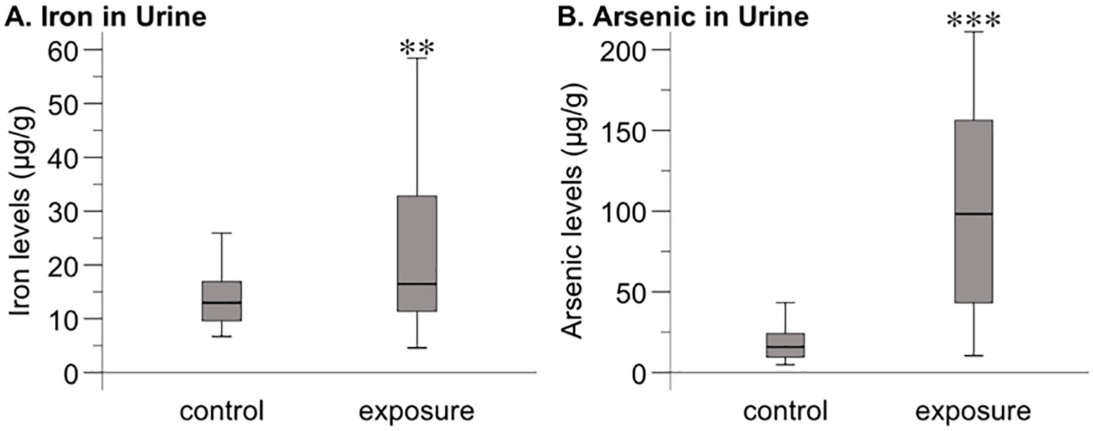

Table S1. Odds ratios (95% CI) of hearing loss in the high iron group at 4 kHz.

	Iron (μg/g)	**Model 1**	**Model 2**
Low	≤ 40.9	Reference	Reference
High	> 41.0	2.750 (1.052, 7.188)	2.746 (1.028, 7.263)
P value		0.039	0.042

Reference: OR = 1.

Model 1 without any adjustments.

Model 2 was adjusted for age, smoking, and BMI, which have been reported to affect hearing.

Table S2. Classification of low and high iron groups in biological samples.

	Iron (μg/g)	Participants	Percentage (%)
Hair	Low (< 64)	107	73.8
	High (≥ 64)	38	26.2
Toenails	Low (< 280)	95	65.5
	High (≥ 280)	50	34.5

Receiver operating characteristic (ROC) curves and the Youden index were used to determine cut-off values for iron levels in biological samples (Ohgami et al., 2018).

Table S3. Odds ratios (95% *CI*) of hearing loss in the high iron group without and with adjustment for arsenic in biological samples.

		Hearing loss			
		1 kHz (≥ 10 dB)	4 kHz (≥ 10 dB)	8 kHz (≥ 25 dB)	12 kHz (≥ 40 dB)
**Iron in hair**					
Model 1	Low/low	Reference	Reference	Reference	Reference
	High/low	0.899 (0.363, 2.438)	3.921** (1.463, 10.507)	4.341** (1.584, 11.897)	3.409* (1.308, 8.881)
	P value	0.899	0.007	0.004	0.012
Model 2	Low/low	Reference	Reference	Reference	Reference
	High/low	0.965 (0.372, 2.504)	3.921** (3.874, 10.296)	4.188** (1.542, 11.375)	3.112* (1.176, 8.237)
	P value	0.941	0.007	0.005	0.022
**Iron in toenails**					
Model 1	Low/low	Reference	Reference	Reference	Reference
	High/low	0.858 (0.331, 2.223)	2.496* (1.009, 6.173)	3.044* (1.178, 7.866)	3.025* (1.217, 7.517)
	P value	0.752	0.048	0.022	0.017
Model 2	Low/low	Reference	Reference	Reference	Reference
	High/low	0.833 (0.333, 2.345)	2.829* (1.082, 7.399)	2.897* (1.079, 7.772)	2.171 (0.830, 5.680)
	P value	0.804	0.034	0.035	0.114

Abbreviation: CI, confidence interval.

Reference: OR = 1.

Model 1 was adjusted for age, gender, smoking, and BMI, which have been reported to affect hearing.

Model 2 was further adjusted for arsenic based on Model 1.

P value was determined by binary logistic regression analysis, $^{*}P < 0.05$, $^{**}P < 0.01$

Table S4. Nagelkerke R square of logistic regression model and relative contribution of each variable to hearing loss at each frequency.

	Hearing loss							
	1 kHz (≥ 10 dB)		4 kHz (≥ 10 dB)		8 kHz (≥ 25 dB)		12 kHz (≥ 40 dB)	
	Hair	Toenail	Hair	Toenail	Hair	Toenail	Hair	Toenail
Pseudo R Square（%）								
Iron	0.04	0.51	10.77	7.32	11.26	6.95	6.98	4.04
Arsenic	5.55	0.69	6.59	1.11	5.77	0.26	24.15	11.9
BMI	7.69	6.06	3.48	3.22	5.53	6.21	0.35	0.58
Age	14.33	20.21	24.23	26.37	22.1	22.98	18.52	25.12
Smoking	0.55	34.52	6.07	7.29	3.38	2.82	0.96	1.02
Gender	27.03	2.22	3.68	12.45	9.03	20.07	0.3	4.35
Redundancy	44.81	35.79	45.18	42.24	42.93	40.71	48.74	52.99
Nagelkerke R square (%)								
Model	12.24	11.73	45.90*	40.76*	47.02*	42.21*	47.75*	40.41*

*In multivariate analyses at 4, 8 and 12 kHz, the models accounted for 40% to 46% of Nagelkerke R Square of the variances, presenting a good overall fit (Wong et al., 2008).

Table S5. Interaction effect of iron and arsenic on hearing loss

	Hearing loss			
	1 kHz (≥ 10 dB)	4 kHz (≥ 10 dB)	8 kHz (≥ 25 dB)	12 kHz (≥ 40 dB)
**Iron and arsenic interaction effect in hair**				
OR	1.543	2.339	3.773	4.133
95% *CI*	0.504, 4.723	0.283, 19.321	0.363, 39.207	0.321, 53.162
P value	0.386	0.430	0.266	0.276
**Iron and arsenic interaction effect in toenails**				
OR	0.377	1.244	0.337	0.371
95% *CI*	0.021, 6.630	0.063, 24.712	0.017, 6.482	0.020, 7.057
P value	0.505	0.886	0.471	0.510

The same models as those shown in Table S3 were used.

Abbreviation: CI, confidence interval.

Reference: OR = 1.

Table S6. Odds ratios (95% *CI*) of hearing loss in the exposure group*.

	Hearing loss			
	1 kHz (≥ 10 dB)	4 kHz (≥ 10 dB)	8 kHz (≥ 25 dB)	12 kHz (≥ 40 dB)
Control	Reference	Reference	Reference	Reference
Exposure	0.505 (0.086, 2.966)	1.509 (0.302, 7.544)	4.701 (0.802, 27.558)	2.525 (0.389, 16.372)
P value	0.449	0.616	0.086	0.331

*Male subjects in the exposure group (n=32) and the control group (n=37) in Fig. S4 were analyzed.

Abbreviation: CI, confidence interval.

Reference: OR=1.

Adjustments were made for age, smoking, BMI and arsenic in urine samples, which have been reported to affect hearing.

P value was determined by binary logistic regression analysis.
